# Hepatitis B virus X protein up-regulates C4b-binding protein α through activating transcription factor Sp1 in protection of hepatoma cells from complement attack

**DOI:** 10.18632/oncotarget.8472

**Published:** 2016-03-30

**Authors:** Guoxing Feng, Jiong Li, Minying Zheng, Zhe Yang, Yunxia Liu, Shuqin Zhang, Lihong Ye, Weiying Zhang, Xiaodong Zhang

**Affiliations:** ^1^ State Key Laboratory of Medicinal Chemical Biology, Department of Cancer Research, College of Life Sciences, Nankai University, Tianjin, P.R. China; ^2^ State Key Laboratory of Medicinal Chemical Biology, Department of Biochemistry, College of Life Sciences, Nankai University, Tianjin, P.R. China

**Keywords:** HBx, C4BP, complement attack, HCC

## Abstract

Hepatitis B virus X protein (HBx) plays crucial roles in the development of hepatocellular carcinoma (HCC). We previously showed that HBx protected hepatoma cells from complement attack by activation of CD59. Moreover, in this study we found that HBx protected hepatoma cells from complement attack by activation of C4b-binding protein α (C4BPα), a potent inhibitor of complement system. We observed that HBx were positively correlated with those of C4BPα in clinical HCC tissues. Mechanistically, HBx activated the promoter core region of C4BPα, located at −1199/−803nt, through binding to transcription factor Sp1. In addition, chromatin immunoprecipitation (ChIP) assays showed that HBx was able to bind to the promoter of C4BPα, which could be blocked by Sp1 silencing. Functionally, knockdown of C4BPα obviously increased the deposition of C5b-9, a complex of complement membrane attack, and remarkably abolished the HBx-induced resistance of hepatoma cells from complement attack *in vitro* and *in vivo*. Thus, we conclude that HBx up-regulates C4BPα through activating transcription factor Sp1 in protection of liver cancer cells from complement attack. Our finding provides new insights into the mechanism by which HBx enhances protection of hepatoma cells from complement attack.

## INTRODUCTION

Hepatocellular carcinoma (HCC) is one of the five most common malignancies and the third leading cause of cancer related deaths worldwide [1]. The chronic infection of hepatitis B virus (HBV) is a major risk factor in the development of HCC [2]. HBV-associated HCC is associated with the multifactorial process that largely depends on the efficacy of the host immune response to the virus [3]. Hepatitis B virus X protein (HBx) plays crucial roles in the viral pathogenesis and carcinogenesis [4–6]. It has been reported that HBx was able to bind to various transcription factors or components of signal transduction pathways to stimulate transcription, signal transduction, and cell cycle progress [7]. Our laboratory reported that HBx promoted the development of hepatoma through YAP. HBx was able to up-regulate Lin28A/Lin28B through activation of transcription factor Sp1. HBx up-regulated the oncogene Rab18, resulting in the dysregulation of lipogenesis and proliferation of hepatoma cells. In addition, HBx functionally accelerated hepatocarcinogenesis with its partner survivin [8–11]. Interestingly, we showed that HBx up-regulated CD59 and or CD46 to inhibit complement activation and protected hepatoma cells from complement attack in the development of HCC [12, 13]. However, the mechanism by which HBx induces escape of hepatoma cells from immune surveillance is not clearly documented.

The complement system displays a central role in innate immunity and serves as a highly effective way for the destruction of invading microorganisms and for immune complex elimination [14]. It defends the host against infections, bridges innate and adaptive immunity and disposes of immune complexes and apoptotic cells. Complement activation occurs via three routes, such as the classical, the lectin and the alternative pathway. Irrespective of the pathway involved, the activation of the complement system leads to the cleavage of C3 and C5, generating the potent chemo-attractants C3a and C5a as well as the C3b and C5b fragments. The latter initiates the assembly of the C5b-9 membrane attack complex (MAC), a lipophilic complex which forms pores in cell membranes, leading to cell lysis [15, 16]. Membrane complement regulatory proteins (mCRPs), including CD46, CD55 and CD59, are widely expressed in almost all cell types, and their inhibitory effect on complement activation is extremely important for protecting normal tissues against immunopathology [17]. It has been reported that mCRPs enables tumor cells to evade complement-dependent cytotoxicity (CDC) and antibody-dependent killing mechanisms. MCRPs are involved in various malignant tumors, including ovarian cancer, endometrial cancer, breast cancer, lung cancer, chronic lymphocytic leukemia (CLL) and liver cancer [18–24].

C4b-binding protein (C4BP) are characterized by convertase decay-acceleration activity, an ability to accelerate convertase disassembly, as well as cofactor activity, as they act as cofactors supporting cleavage by factor I (FI) of the activated complement components C3b and or C4b necessary for convertase formation [25]. C4BP has three isoforms with different subunit composition, in which C4BPα and C4BPβ subunits are mainly expressed in the liver. C4BPα has the binding site for C4b. However, C4BPβ is not essential for the binding of C4BPα to C4b. C4BP interferes directly with the formation of C3 convertase and enhances the natural decay of this enzymatic complex [26–28]. In addition, C4BP compensates the down-regulation of mCRPs on apoptotic cells and protects the cells from excessive complement activation and lysis [29]. Cancer cells avoid the immune system attack through disguising themselves from normal counterparts, making them to escape immune recognition or activation [30–32]. However, the mechanism of complement system regulation in liver cancer is not well understood.

In the present study, to better understand the mechanism by which HBx accelerates the protection of liver cancer cells from complement attack, we investigated the effect of HBx on C4BPα in the event. Interestingly, our data indicated that HBx was able to up-regulate C4BPα via activation of transcription factor Sp1 in promoting the resistance of CDC in HCC. Our findings provide new insights into the mechanism of HBx-activated immune escape in the development of HCC.

## RESULTS

### The expression levels of HBx are positively correlated with those of C4BPα in clinical HCC tissues

**Figure 1:**
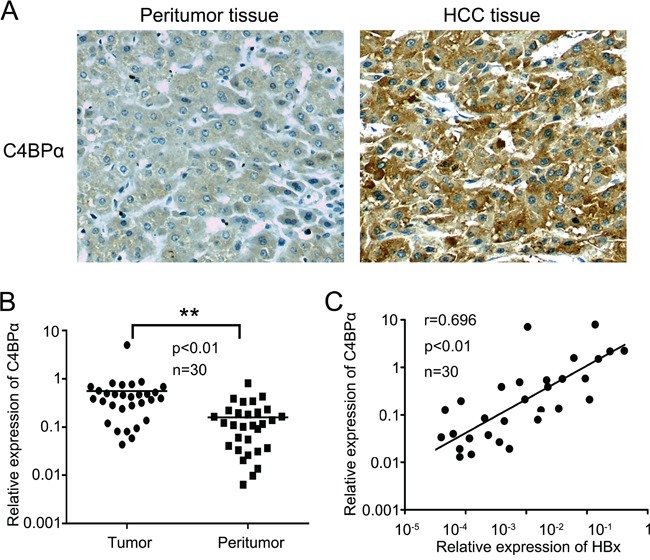
The expression levels of HBx are positively correlated with those of C4BPα in clinical HCC tissues **A.** The expression levels of C4BPα were determined in the liver carcinoma and adjacent normal liver tissues by IHC analysis using tissue microarray. **B.** The relative mRNA expression levels of C4BPα were detected by qRT-PCR analysis in clinical liver carcinoma specimens and adjacent normal tissues (n = 30) (**p<0.01, Wilcoxon signed-rank test). **C.** The correlation of mRNA levels of C4BPα and HBx was examined by qRT-PCR assay in 30 pairs liver carcinoma tissues (*p*<0.01, r=0.696, Pearson's correlation).

According to the reports that HBx-induced resistance of hepatoma cells from complement attack in HCC and expression of C4BP was increased in HBx-transfected hepatic LO2 cells using cDNA microarray analysis [12, 13, 33], we are interested in whether HBx activates C4BP in the protection of hepatoma cells from complement attack in HCC. Then, we evaluated the relationship between HBx and C4BP in clinical liver tissues. Immunohistochemistry (IHC) analysis showed that the positive rate of C4BP was 87.4% (125/143), in which the strong positive rate of C4BP was 30.1% (43/143), in HCC tissues using tissue microarray containing 8 normal liver and 143 liver tumor cores (Figure 1A; Supplementary Table S1). In addition, we tested the expression of C4BP by IHC in formalin-fixed paraffin embedded pellets of breast cancer tissues. Our data showed the expression of C4BP was negative (Figure S1 G-I), suggesting that the specificity of C4BP staining is good. It has been noted that C4BPα has the binding site for C4b, while C4BPβ is not essential for the binding of C4BPα to C4b. Thus, we focused on the investigation of C4BPα in our study. Real-time PCR revealed that the mRNA levels of C4BPα were significantly increased in 30 HCC tissues relative to their adjacent noncancerous tissues (*P* <0.01, Wilcoxon's signed-rank test, Figure 1B). Strikingly, the mRNA levels of HBx were positively correlated with those of C4BPα in above 30 clinical HCC tissues (r = 0.696, *P* < 0.01, Pearson's correlation, Figure 1C), suggesting that HBx might up-regulate C4BPα in hepatoma cells. Therefore, we conclude that the expression of HBx is positively correlated with that of C4BPα in clinical HCC tissues.

### HBx up-regulates C4BPα in hepatoma cells

**Figure 2:**
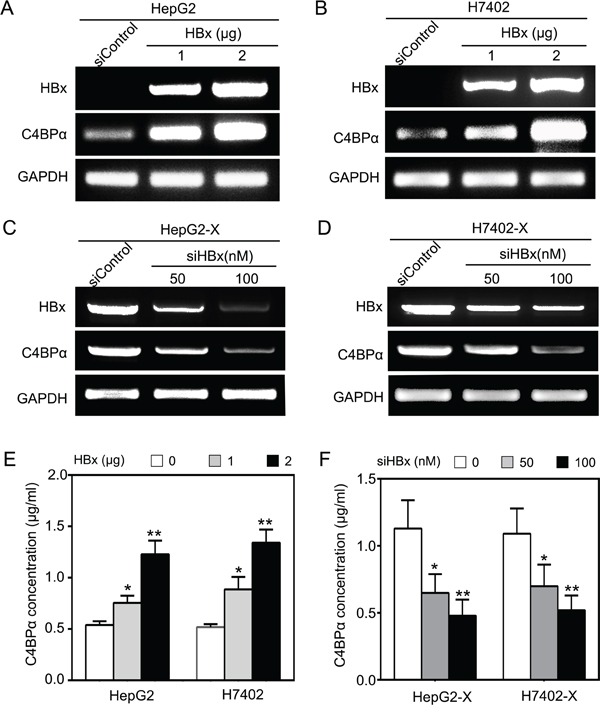
HBx up-regulates C4BPα in hepatoma cell **A, B.** The mRNA levels of HBx and C4BPα were tested by RT-PCR analysis in HepG2 (or H7402) cells transiently transfected with pCDNA3.1-HBx. **C, D.** The expression levels of HBx and C4BPα were examined by RT-PCR analysis in HepG2-X cells (or H7402-X) treated with HBx siRNA. **E.** The protein levels of C4BP were examined by ELISA in the supernatant of HepG2 (or H7402) cells treated with pCDNA3.1-HBx. **F.** The protein levels of C4BP were examined by ELISA in the supernatant of HepG2-X (or H7402-X) cells treated with HBx siRNA. *p<0.05, **p<0.01, Student's *t*-test. All experiments were performed at least three times.

Next, we attempted to validate the effect of HBx on C4BPα in hepatoma cells. Our data demonstrated that the overexpression of HBx was able to up-regulate C4BPα at the levels of mRNA in hepatoma HepG2 and H7402 cells in a dose-dependent manner (Figure 2A and 2B). Moreover, HBx small interference RNA (siRNA) significantly decreased the mRNA levels of C4BPα in HepG2-X/H7402-X cells with stably expression of HBx (Figure 2C and 2D). According to the report that C4BPα is a plasma glycoprotein [34], we further tested the levels of C4BPα in the conditioned media of hepatoma cells. ELISA assays showed that the expression levels of C4BPα protein were markedly increased in the conditioned media of HepG2/H7402 cells treated with HBx in a dose-dependent manner. In contrast, the expression levels of C4BPα protein were decreased in HepG2-X/H7402-X cells treated with HBx siRNA. Thus, we conclude that HBx is able to up-regulate C4BPα in hepatoma cells.

### HBx is able to activate the core region −1199/−803nt in promoter of C4BPα

**Figure 3:**
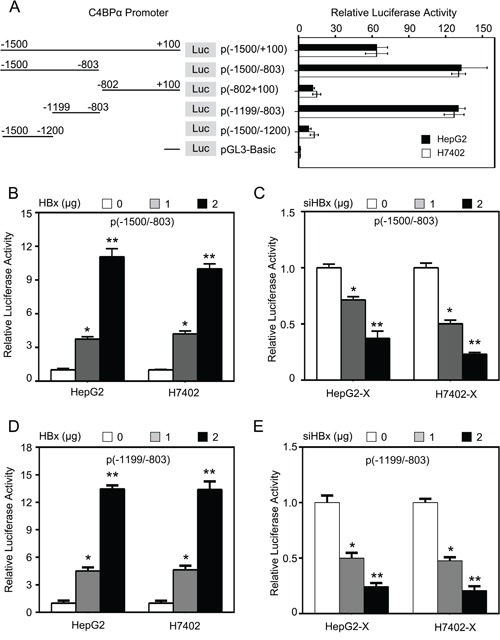
HBx is able to activate the core region −1199/−803nt in promoter of C4BPα **A.** The relative activities of different regions of C4BPα promoter were examined by luciferase reporter gene assays in HepG2 (or H7402) cells. **B.** The relative activities of the core promoter (−1500/−803nt) of C4BPα were detected by luciferase reporter gene assays in HepG2 cells or H7402 cells transiently transfected with pCDNA3.1-HBx. **C.** The relative activities of the core promoter (−1500/−803nt) of C4BPα were detected by luciferase reporter gene assays in HepG2-X cells (or H7402-X) transiently transfected with HBx siRNA. **D.** The relative activities of the core promoter (−1199/−803nt) of C4BPα were detected by luciferase reporter gene assays in HepG2 cells or H7402 cells transiently transfected with pCDNA3.1-HBx. **E.** The relative activities of the core promoter (−1199/−803nt) of C4BPα were detected by luciferase reporter gene assays in HepG2-X cells (or H7402-X) transiently transfected with HBx siRNA. *p<0.05, **p<0.01, Student's *t*-test. All experiments were performed at least three times.

To identify the mechanism by which HBx up-regulates C4BPα, we examined the effect of HBx on promoter of C4BPα in hepatoma cells. To screen the core region in promoter of C4BPα, we cloned the different fragments of C4BPα 5′-flanking region, including -1500/+100nt (pGL3-C4BP-P1), −1500/−803nt (pGL3-C4BP-P2), -802/+100nt (pGL3-C4BP-P3), −1199/−803nt (pGL3-C4BP-P4) and −1500/−1200nt (pGL3-C4BP-P5). Then, the plasmids were transiently transfected into the HepG2 and H7402 cells. The luciferase reporter gene assays indicated that the vector of pGL3-C4BP-P4 containing fragment −1199/−803nt exhibited the maximum luciferase activities (Figure 3A), suggesting that the fragment might be the core region of C4BPα promoter. Then, we examined the effect of HBx on C4BPα promoter. The results indicated that HBx was able to activate the luciferase activities of pGL3-C4BP-P2 in HepG2 and H7402 cells in a dose-dependent manner (Figure 3B). In contrast, HBx siRNA abolished the luciferase activities of pGL3-C4BP-P2 in HepG2-X and H7402-X cells in a dose-dependent manner (Figure 3C). Moreover, HBx was able to activate the luciferase activities of pGL3-C4BP-P4 in HepG2 and H7402 cells in a dose-dependent manner (Figure 3D). In contrast, HBx siRNA abolished the luciferase activities of pGL3-C4BP-P4 in HepG2-X and H7402-X cells in a dose-dependent manner (Figure 3E). Thus, we conclude that HBx is able to activate the core region −1199/−803nt in promoter of C4BPα.

### HBx activates C4BP promoter through transcription factor Sp1

**Figure 4:**
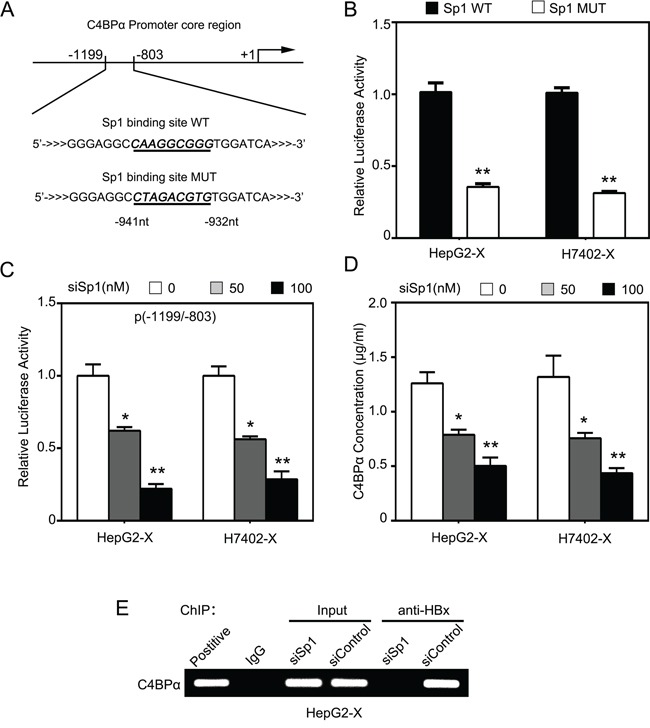
HBx activates C4BPα promoter through transcription factor Sp1 **A.** The illustration of the responsive region to HBx in C4BPα promoter was shown. **B.** The relative activities of the core promoter (−1199/−803nt) of C4BPα containing wild type (WT) or mutant type (MUT) of Sp1 binding site were detected by luciferase reporter gene assays in HepG2-X or (H7402-X) cells. **C.** The relative activities of the core promoter (−1199/−803nt) of C4BPα were detected by luciferase reporter gene assays in HepG2-X or (H7402-X) cells transiently transfected with Sp1 siRNA. **D.** The protein levels of C4BPα were examined by ELISA in the supernatant of HepG2-X (or H7402-X) cells treated with Sp1 siRNA. **E.** The interaction between HBx and Sp1 in HepG2-X cells was examined by ChIP assays. *p<0.05, **p<0.01, Student's *t*-test. All experiments were performed at least three times.

Next, we predicted the transcription factor binding sites in the core region −1199/−803nt of C4BPα promoter using WWW Promoter Scan (http://www-bimas.cit.nih.gov/molbio/proscan/). Interestingly, we observed that there were many different binding elements of transcription factors in the promoter region −1199/−803nt of C4BPα, such as Sp1 (Figure 4A). We previously reported that HBx activated Lin28A/Lin28B through Sp1/c-Myc in hepatoma cells [8]. Therefore, we hypothesized that HBx might stimulate C4BPα promoter through activation of transcription factor Sp1. Interestingly, the promoter activities of C4BPα could be abolished when the binding site of Sp1 in C4BPα promoter was mutated in HepG2-X and H7402-X cells (Figure 4B). Furthermore, the elevated promoter activities of C4BPα induced by HBx could be disrupted by Sp1 siRNA in the cells in a dose-dependent manner (Figure 4C), suggesting that HBx might activate C4BPα promoter through transcription factor Sp1. ELISA assays further validated that Sp1 siRNA could block the HBx-increased expression levels of C4BPα in the supernatant of HepG2-X and H7402-X cells (Figure 4D). Meanwhile, the RNA interference efficiency was validated by Western blot analysis in the cells (Figure S2). We previously reported that HBx was able to transactivate Sp1 to activate transcription of Lin28A/Lin28B [8, 10]. Our data validated that Sp1 could be immunoprecipitated by anti-HBx antibody in the cells and vice versa (data not shown). Moreover, chromatin immunoprecipitation (ChIP) assays showed that HBx was able to bind to the fragment −1199/−803nt of C4BPα promoter in HepG2-X cells. Interestingly, Sp1 siRNA abolished the binding of HBx to the region (Figure 4E). Taken together, we conclude that HBx activates C4BPα promoter through transcription factor Sp1.

### Down-regulation of C4BPα increases the sensitivity of hepatoma cells to CDC

**Figure 5:**
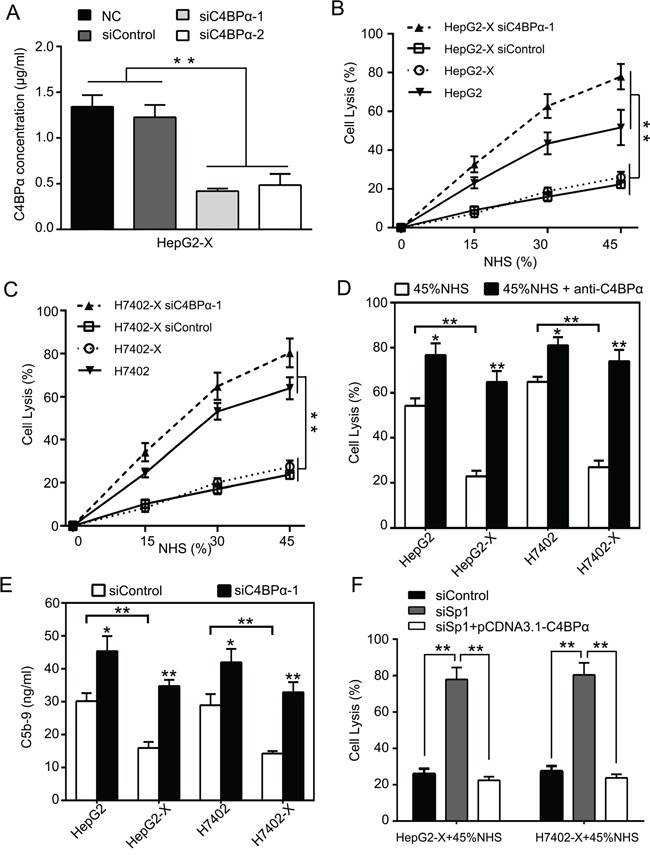
Down-regulation of C4BPα increases the sensitivity of hepatoma cells to CDC **A.** The efficiency of C4BPα siRNA was examined by ELISA in the supernatant of HepG2-X cells. **B, C.** The effect of C4BPα siRNA on CDC was determined by Trypan blue absorbance assays in HepG2, HepG2-X (or H7402, H7402-X) cells treated with different dilutions of normal human serum. **D.** The effect of antibody against C4BPα on CDC was examined by Trypan blue absorbance assay in HepG2, HepG2-X (or H7402, H7402-X) cells treated with 45% of normal human serum. **E.** The deposition of C5b-9 was measured by ELISA in HepG2, HepG2-X (or H7402, H7402-X) cells treated with C4BPα siRNA. **F.** The effect of Sp1 siRNA or overexpression of C4BPα on CDC was examined by Trypan blue absorbance assays in HepG2-X (or H7402-X) cells treated with 45% of normal human serum. *p<0.05, **p<0.01, Student's *t*-test. All experiments were performed at least three times.

Next, we examined the effect of C4BPα on protection of hepatoma cells from complement attack activated by HBx. We previously reported that HBx activated CD59 in protection of hepatoma cells from complement attack [12]. Accordingly, we determined the effect of C4BPα on the complement-dependent cytotoxicity (CDC) in HepG2-X and H7402-X cells. We first evaluated the efficiency of RNA interference for C4BPα using ELISA assays in HepG2-X cells (Figure 5A). CDC assays showed that the silencing of C4BPα using siC4BPα-1 resulted in the increased sensitivity of HepG2-X (or H7402-X) cells to CDC relative to HepG2 (or H7402) cells (Figure 5B and 5C). Moreover, the addition of C4BPα antibody led to the enhanced sensitivity of HepG2-X (or H7402-X) cells and HepG2 (or H7402) cells to CDC (Figure 5D), supporting that C4BPα contributes to the escape of hepatoma cells from complement attack induced by HBx. It has been reported that C5b-9 membrane attack complex (MAC) is involved in the cell lysis [35]. Therefore, we evaluated the effect of C4BPα on deposition of C5b-9 in hepatoma cells by ELISA assays. Our data showed that the deposition of C5b-9 was decreased on the surface of HepG2-X compare to HepG2 (or H7402-X to H7402) cells. In contrast, the deposition of C5b-9 was increased by C4BPα siRNA on the surface of HepG2 and HepG2-X (or H7402 and H7402-X) cells (Figure 5E; Figure S3). In addition, we validated that Sp1 siRNA increased the sensitivity of HepG2-X and H7402-X cells to CDC using CDC analysis, whereas overexpression of C4BPα could abolish the increase (Figure 5F), supporting that Sp1 is involved in the escape of hepatoma cells from complement attack induced by C4BPα. Collectively, we conclude that down-regulation of C4BPα increases the sensitivity of hepatoma cells to CDC.

### SiC4BPα results in suppression of growth of hepatoma cells through inducing sensitivity of hepatoma cells to CDC *in vivo*

**Figure 6:**
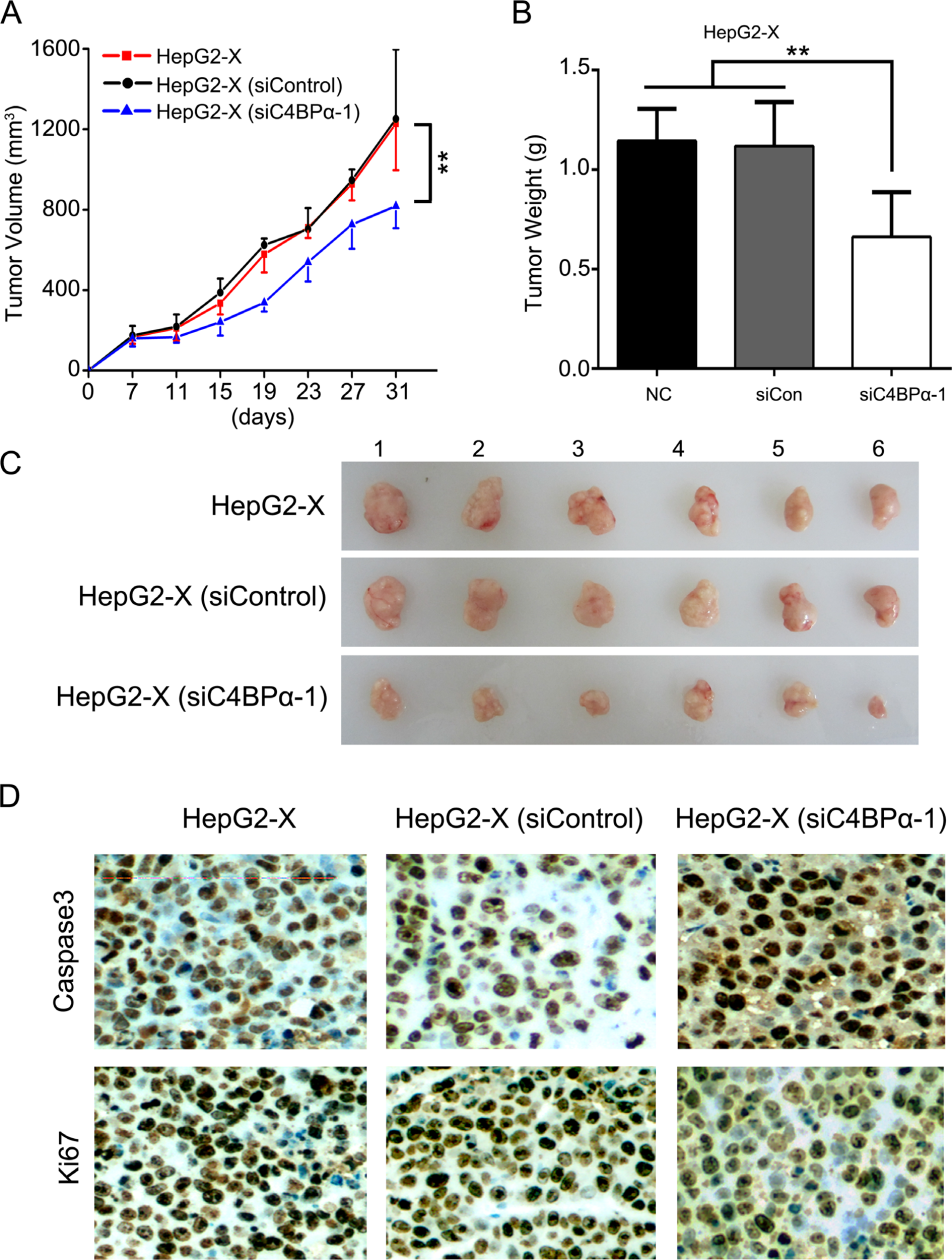
SiC4BPα results in suppression of growth of hepatoma cells through inducing sensitivity of hepatoma cells to CDC *in vivo* **A, B.** HepG2-X cells treated with C4BPα siRNA were transplanted into the nude mice. The growth curve and average weight of tumors were shown. **C.** The image of dissected tumors from nude mice (n = 6). **D.** The expression of caspase3 and Ki67 was tested by IHC analysis in the tumor tissues from nude mice. *p<0.05, **p<0.01, Student's *t*-test.

Given that HBx-elevated CD59 depressed the growth of hepatoma cells through protection of hepatoma cells from complement attack [12, 13]. Then, we further investigated the effect of C4BPα on the growth of hepatoma cells *in vivo*. Our data demonstrated that the volume and weight of the tumor were significantly lower by the treatment with siC4BPα in mice (Figure 6A–6C). It has been reported that the complement system leads to insertion of terminal complement complexes (C5b-9) into the cell membrane, which may induce cytolysis. C5b-9 is involved in induction of apoptosis via a caspase-dependent pathway [35]. Accordingly, we evaluated the levels of caspase3, an apoptosis biomarker, in the tumor tissues from mice. Interestingly, the expression of caspase3 was increased in HepG2-X group treated with siC4BPα relative to controls (Figure 6D). Meanwhile, the expression levels of Ki67, a cell proliferation marker, were also significantly decreased in the system (Figure 6D), suggesting that siC4BPα induces apoptosis and depresses the proliferation of hepatoma cells. Thus, we conclude that siC4BPα is able to suppress the growth of hepatoma cells through inducing sensitivity of hepatoma cells to CDC *in vivo*.

## DISCUSSION

Abundant evidence strongly indicates that HBx contributes to the hepatocarcinogenesis [36–38]. The complement system displays a central role in innate immunity and bridges innate and adaptive immune responses in the development of liver cancer [39]. C4BP is mainly expressed in liver and plays an important role of regulatory of complement balance [39]. We previously found that HBx increased the complement regulation proteins such as CD46 and CD59 to protect hepatoma cells from complement attack [12, 13]. In this study, we are interested in whether HBx stimulates C4BP in protection of hepatoma cells from complement attack.

Given that the expression of C4BP was up-regulated in HBx-transfected hepatic LO2 cells using cDNA microarray analysis [33]. Therefore, we supposed that C4BP might be involved in the escape of hepatoma cells from immune surveillance activated by HBx. We found that HBx increased C4BPα through activating transcription factor Sp1 in protection of liver cancer cells from complement attack in a nude mouse model. In addition, siC4BPα markedly inhibited depositions of C5b-9 on the cell surface and caused a significant CDC in the HepG2-X or H7402-X cells. These results confirm that C4BPα maintains its functional attributes, which were initially gleaned from *in vitro* studies [40], in a complementary immunocompetent xenograft HCC model.

It has been reported that C4BP has three isoforms with different subunit composition. Functionally, C4BPα has the binding site for C4b, however, C4BPβ is not essential for the binding of C4BPα to C4b. Thereby, we focused on the investigation of C4BPα in our system. Interestingly, real-time PCR showed the expression levels of C4BPα were elevated in HCC tissues relative to their adjacent noncancerous tissues. Thus, we validated that HBx up-regulated C4BPα in hepatoma cells. It suggests that C4BPα might be involved in the escape of hepatoma cells from immune surveillance induced by HBx.

Next, we sought to identify the underlying mechanism by which HBx up-regulated C4BPα in hepatoma cells. We investigated the effect of HBx on the promoter activities of C4BPα in HBx-positive cell lines. It has been reported that HBx does not directly bind to DNA, it functions through protein–protein interaction [41]. To demonstrate whether HBx activate the C4BPα promoter in the cells, we identified that the fragment −1199/−803nt was the core region of C4BP promoter. Interestingly, we revealed that HBx was able to activate the fragment −1199/−803nt of C4BP promoter. Then, we predicted that the region contained one binding site of transcription factor Sp1. Sp1 is a member of the Sp/KLF family of transcription factors and many genes encoding various cell growth regulatory proteins contain Sp1 sites in their promoters [42]. Sp1 is overexpressed in a variety of cancers including liver, breast, colon, pancreas, bladder and prostate cancer, and plays an important role in cell proliferation by activating the expression of several cell cycle regulatory proteins [43]. Interestingly, we have reported that HBx activates Lin28A/Lin28B through Sp1/c-Myc in hepatoma cells [8]. Then, we validated that the transcription factor Sp1 might be involved in the activation of C4BP promoter stimulated by HBx in hepatoma cells. Our finding is consistent with our report that HBx was able to activate Sp1 in up-regulation of Lin28A/Lin28B in hepatoma cells [8]. Thus, we conclude that HBx is able to up-regulate C4BP through transcription factor Sp1 in hepatoma cells.

It has been reported that C4BP as the key inhibitor disrupts the formation of C3 convertase and enhances the natural decay of this enzymatic complex in the complement activation [26–28, 44]. HBx is involved in the modulation of the immune response [12, 13]. Accordingly, we are interested in whether C4BPα contributes to the HBx-enhanced resist of hepatoma cells to CDC. Interestingly, our data showed that HBx decreased the sensitivity of hepatoma cells and the depositions of C5b-9 on the cell surface. It has been reported that C5b-9 is involved in induction of apoptosis via a caspase-dependent pathway. Interestingly, we observed that the expression of caspase3 was increased in HepG2-X cells treated with siC4BPα relative to controls. It suggests that C4BPα contributes to the HBx-enhanced cell avoiding CDC *in vivo*. This finding is consistent with our report that HBx inhibits complement activation in nude mice, protecting hepatoma cells from complement attack in liver cancer through up-regulating CD46 and CD59 [12, 13]. Thus, we conclude that HBx is able to attenuate the sensitivity of hepatoma cells and hepatic cells to CDC through C4BPα. Therapeutically, HBx may serve as a target in HBV-related liver cancer.

In summary, in this study we report a novel mechanism by which HBx protects hepatoma cells from complement attack through activation of C4BPα. Our data show that HBx is able to up-regulate C4BPα through activation of transcription factor Sp1 in the promoter of C4BPα. C4BPα is involved in the escape of hepatoma cells from complement attack induced by HBx. Thus, our finding provides further insights into the mechanism by which HBx increases resistance of complement attack involving the complement system in the development of HCC.

## MATERIALS AND METHODS

### Patient samples

Thirty pairs of liver cancer tissues with their paratumor tissues utilized in this study were immediately obtained from Tianjin Cancer Hospital (Tianjin, China) after surgical resection. Written consents approving the use of their tissues for research purposes after the operation were obtained from each patient. The study protocol was approved by the institute research ethics committee at Nankai University (Tianjin, China).

### Cell lines and cell culture

Liver cancer cell lines, HepG2, HepG2-X (a stable HBX transfected cell line of HepG2), H7402, H7402-X (a stable HBX transfected cell line of H7402) [45, 46], were cultured in DMEM medium (Gibco, Grand Island, NY), 10% fetal calf serum (FCS), 100 U/ml penicillin, and 100 μg/ml streptomycin in humidified 5% CO2 at 37°C. HepG2-X and H7402-X maintained with G418 200 μg/ml.

### Plasmid construction and small interference RNA (siRNA)

The full-length C4BPα was cloned into the pcDNA3.1 vector to generate the pcDNA3.1-C4BPα. The 5′-flanking region (from -1500 to + 100nt) of C4BPα gene was inserted into the NheI/HindIII site in the upstream of the luciferase gene in the pGL3-Basic vector, including -1500/+100nt (pGL3-C4BP-P1), −1500/−803nt (pGL3-C4BP-P2), -802/+100nt (pGL3-C4BP-P3), −1199/−803nt (pGL3-C4BP-P4) and −1500/−1200nt (pGL3-C4BP-P5). Mutant of C4BPα promoter carried a series substitution of nucleotides within Sp1 binding site was constructed. SiRNAs duplexes targeting HBx, C4BPα and negative control (NC) were synthesized by RiboBio (Guangzhou, China). All primers and siRNA sequence were listed in Supplementary Table S2.

### RNA extraction, RT-PCR and real-time PCR

Total RNA of cells (or liver cancer tissues from patient tumors) was extracted using Trizol reagent (Takara, Dalian, China). First-strand cDNA was synthesized by Transcriptor First Strand cDNA Synthesis Kit (Roche, Mannheim, Germany) following the manufacturer's instructions. To examine the mRNA levels of C4BPα and HBx, real-time PCR was performed by a FastStart Universal SYBR Green Master (ROX) Kit (Indianapolis, IN, USA) according to the manufacturer's instructions. Double-stranded DNA specific expression was tested by the comparative Ct method using 2^−ΔΔCt^ [47]. All primers were listed in Supplementary Table S2.

### Western blot analysis

Western blot analysis was carried out with previously protocols [48]. Primary antibodies were rabbit anti-Sp1 (Proteintech Group, USA), mouse anti-HBx (Santa Cruz Biotechnology, USA) and mouse anti-β-actin (Sigma, Aldrich, St. Louis, MO, USA). All experiments were repeated at least 3 times.

### Luciferase reporter gene assays

For luciferase reporter gene assays, the liver cancer cells were transfected with plasmids encoding pcDNA3.1-HBx (or siRNAs of HBx) by lipofectamine 2000. The luciferase activities were determined 48 h after transfection, and the results are the average of 3 independent repeats. The luciferase activities in the cell lysates were measured by a dual luciferase reporter assay kit (Promega, Madison, USA), and the luciferase activities were normalized to Renilla luciferase activity. pRL-TK, pGL3-Basic vectors (Promega).

### Chromatin immunoprecipitation assays

The chromatin immunoprecipitation assays (ChIP) was performed using the EpiQuikTM chromatin immunoprecipitation kit from Epigentek Group Inc according to the published methods. Protein-DNA complexes were immunoprecipitated with HBx antibodies, with mouse IgG as a negative control antibody and anti-polymerase II as positive control. DNA collected by these antibodies was subjected to PCR analysis, followed by sequencing. Amplification of soluble chromatin prior to immunoprecipitation was used as an input control. All primers were listed in Supplementary Table S2.

### Co-immunoprecipitation

HepG2-X cells (2× 10^6^) were harvested and lysed in a lysis buffer (20 mM Tris–HCl pH 7.5, 150 mM NaCl, 20 mM KCl, 1.5 mM MgCl_2_, 0.5% NP-40, 1 mM protease inhibitor PMSF, 15% glycerol). The lysates were incubated with anti-HBx or anti-Sp1 antibody and protein G conjugated agarose beads at 4°C for 3 h. The precipitates were washed six times with ice-cold lysis buffer, resuspended in phosphate-buffered saline (PBS), and resolved by SDS–PAGE followed by Western blot analysis.

### Complement-dependent cytotoxicity assays

Complement-dependent cytotoxicity assays were performed using Trypan blue absorbance assays [49]. The above cells were planted onto 24-well plates (1× 10^5^ cells per well). Following overnight incubation, the media was removed, and cells were exposed either to normal human serum (NHS) in different dilutions (15, 30 and 45%) or to an anti-C4BPα antibody (Abcam, Cambridge, USA) that blocks function (10 μg/ml) in the presence of NHS to a final concentration of 45%. After 1h incubation, a 0.4% solution of Trypan blue was added to each well to a final concentration of 0.05% and further incubated at 37°C for 15 min. Next, the medium was removed, and the cells were gently washed with ice-cold PBS (3× 750 μl). The cells were then lysed with 200 μl of 1% sodium dodecyl sulfate (SDS) and gently triturated. Finally, 175 μl of the SDS/Trypan blue solution was transferred to a 96-well plate, and the absorbance was read with a Multiskan Ascent enzyme-linked immunosorbent assay (ELISA) reader (Thermo Electron Corporation, Dreieich, Germany) at 590 nm as reported previously [50]. Each test was assayed in triplicate.

### Enzyme-linked immunosorbent assays (ELISA)

The amounts of C5b-9 (a product of functional MAC) were determined by human C5b-9 ELISA kit (RD Scientific, Minneapolis, MN) according to the manufacturer's instructions. The cells were incubated in 6-well plates (2× 10^5^ cells each) for 12 h. After treatment, the cells were washed with PBS three times and incubated with 45% NHS or 45% C5-depleted NHS as a negative control for 2 h. The cells were then lysed as reported previously [12].

### Tumor formation in nude mice

Experimental procedures were performed in accordance with the Guide for the Care and Use of Laboratory Animals (NIH Publication No. 80-23, revised 1996) and were performed according to the institutional ethical guidelines for animal experiments. BALB/c nude mice were employed for tumorigenicity analysis. The tumorigenicity of HepG2-X cells were measured as reported previously. Cells were harvested by trypsinization, washed twice with sterile PBS and resuspended at 2× 10^7^ cells/ml. Then, 0.2 ml aliquots were injected subcutaneously into 4-week-old female BALB/c nude mice with healthy complement system (n = 6, in each group). The mice received HepG2-X cells pretreated with siRNA Ctronl or C4BPα siRNA were observed at periodic intervals. Tumor growth was measured after 7 days from injection and then every 4 days. At 30 days after injection, mice were killed and tumors were weighted after necropsy. Tumor volume (V) was monitored by measuring the length (L) and width (W) with calipers and calculated with the formula (L× W^2^)/2.

### Immunohistochemistry (IHC)

Liver cancer tissue array (No. 08C03), comprising 149 liver tumors, was purchased from Xi'an Aomei Biotechnology (Xi'an, China). Breast cancer tissue were immediately obtained from Tianjin Cancer Hospital (Tianjin, China) after surgical resection. IHC staining assay was performed as described previously [8]. The slides were incubated with rabbit anti-C4BPα antibody (1:250 dilution Proteintech Group, Chicago, USA), anti-Ki67 antibody (1:200 dilution, Proteintech Group, Chicago, USA), anti-caspase3 antibody (1:200 dilution, Proteintech Group, Chicago, USA) at 4°C for overnight. Then, the slides were incubated with horseradish peroxidase labelled secondary antibody for 30 min at room temperature. Immunostaining was developed by using chromogen 3, 3′-Diaminobenzidine (DAB), and counterstained with Mayer's hematoxylin (ZSBG-BIO, Beijing, China). The staining levels of C4BP were classified into three groups using a modified scoring method based on the intensity of staining (− negative; + low; ++Middle; +++ high; ++++super high).

### Statistical analysis

Data were analyzed using SPSS software v.13.0 (Chicago, IL). Each experiment was repeated at least three times. The expression levels of C4BPα in liver cancer tissues and their corresponding nontumorous tissues were analyzed using the Wilcoxon signed-rank test. Pearson's correlation coefficient was used to determine the correlation between HBx and C4BPα in clinical HCC tissues. Statistical significance was assessed by comparing the mean values (±SD) using a Student's *t*-test.

## SUPPLEMENTARY FIGURES AND TABLES




