# Supplementary material for: Hepatitis B virus X protein up-regulates C4b-binding protein α through activating transcription factor Sp1 in protection of hepatoma cells from complement attack
Source: Oncotarget. 2016 Mar 30;7(19):28013–26. doi: 10.18632/oncotarget.8472 (PMC5053706; doi:10.18632/oncotarget.8472)
Supplement: Supplementary file 1 [file oncotarget-07-28013-s001.pdf]

Hepatitis B virus X protein up-regulates C4b-binding protein α through activating transcription factor Sp1 in protection of hepatoma cells from complement attack

SUPPLEMENTARY FIGURES AND TABLES

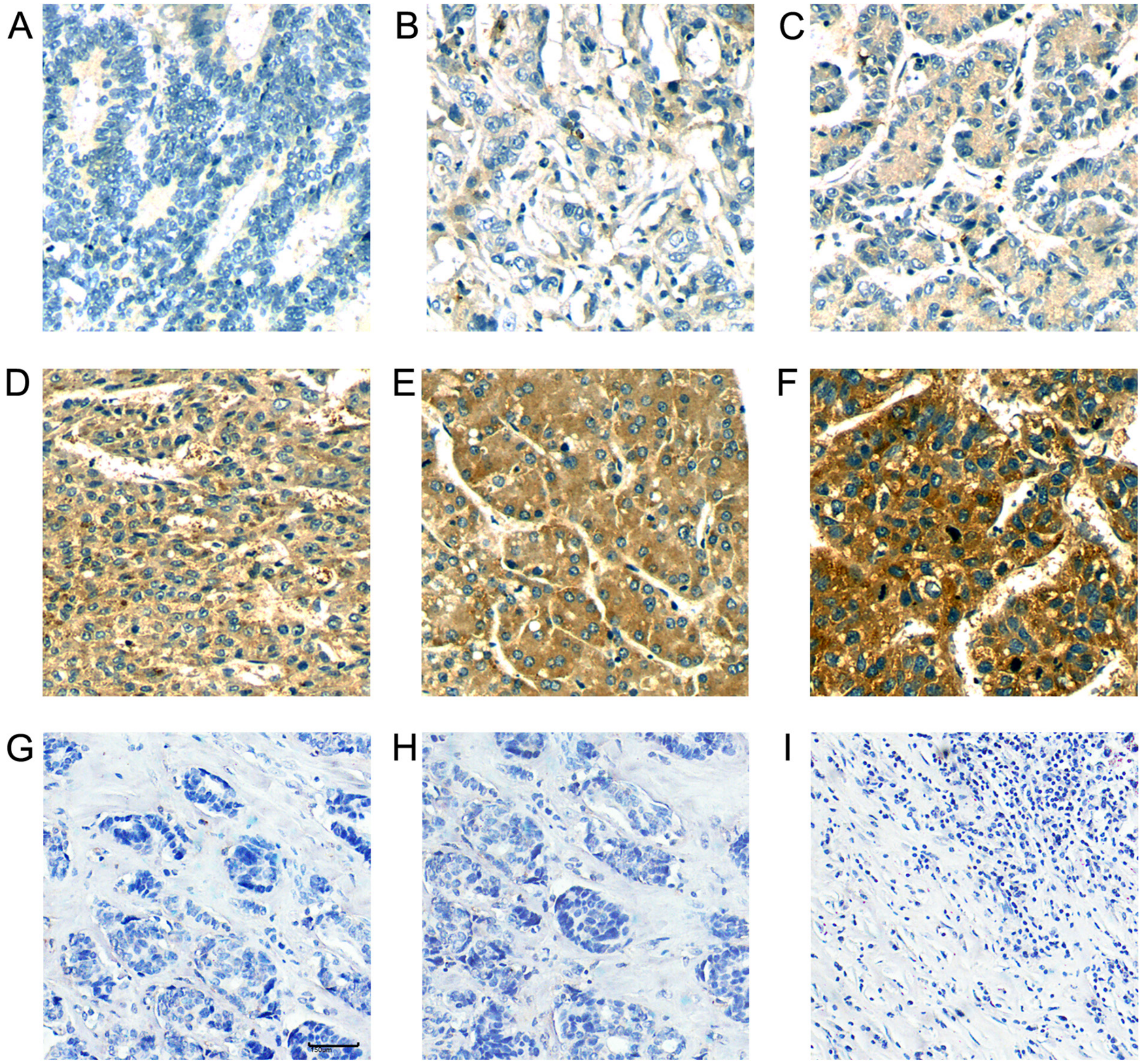

Supplementary Figure S1: The expression levels of HBx are positively correlated with C4BPα in HCC tissues. Related to Figure 1. **A-F.** Expression of C4BP was examined by IHC in HCC tumor tissues using tissue array, showing 6 samples with different staining. **G-I.** Expression of C4BP was examined by IHC in breast cancer tissues using tissue array.

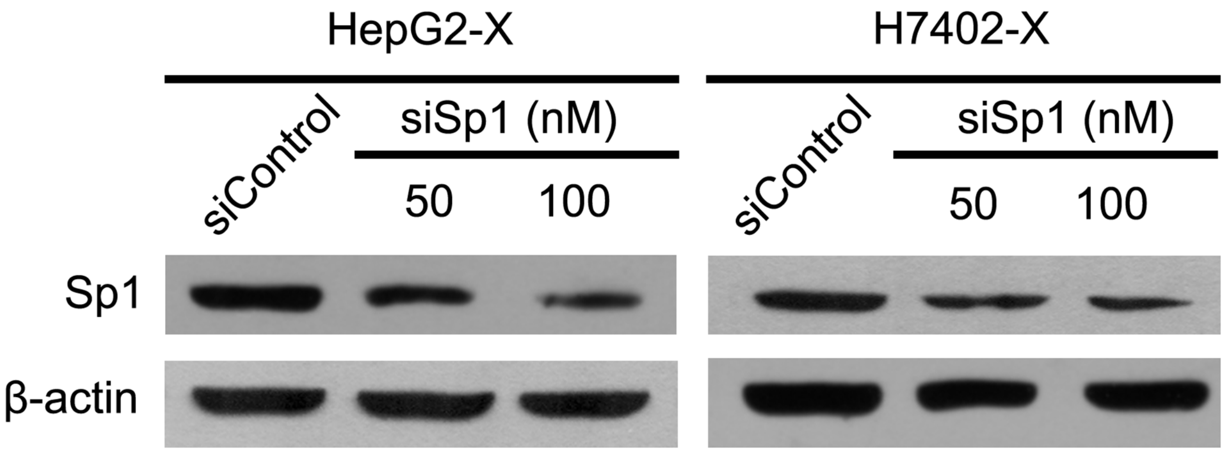

Supplementary Figure S2: HBx activates C4BPα promoter through transcription factor Sp1. Related to Figure 4. The interference efficacy of Sp1 was detected by Western blot analysis in HepG2-X (or H7402-X) cells transfected with Sp1 siRNA.

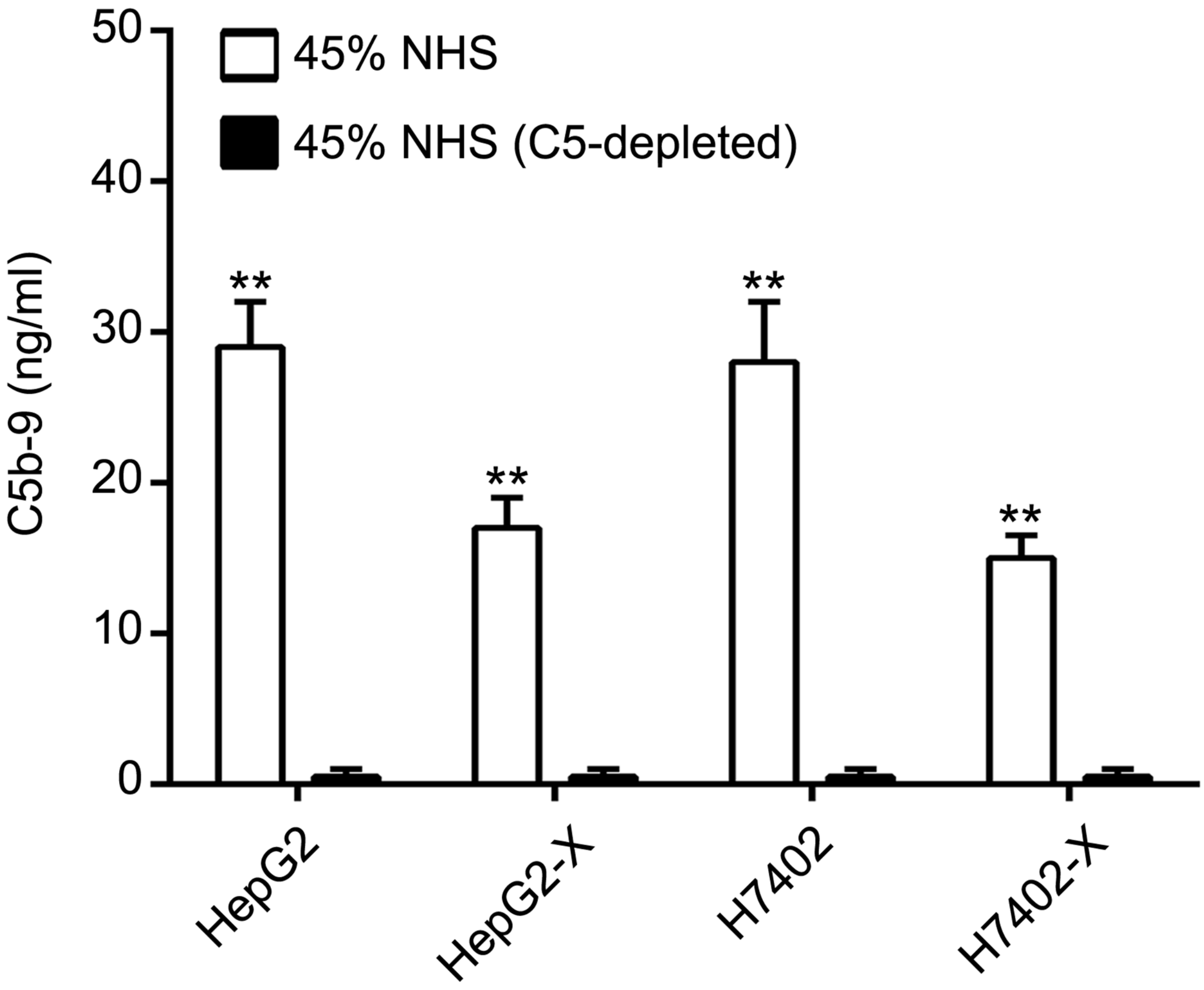

Supplementary Figure S3: Down-regulation of C4BPα increases the sensitivity of hepatoma cells to CDC. **Related to Figure 5.** The effect of C4BPα on deposition of C5b-9 was examined by ELISA assays in HepG2, HepG2-X (or H7402, H7402-X) cells treated with 45% of normal human serum (NHS) or 45% of normal human serum (C5-depleted) (negative control).

Supplementary Table S1: Clinical characteristics of liver cancer from microarray samples.

See Supplementary File 1

Supplementary Table S2: List of primers used in this paper.

Primer	Forward Primer (5′-3′)	Reverse Primer (5′-3′)
**Primers for C4BP promoter**		
-1500/+100nt	CTAGCTAGCTGTCTTAGAGGGTGCTTA	CCCAAGCTTTAAACTAACCT TACCTGT
-1500/-803nt	CTAGCTAGCTGTCTTAGAGGGTGCTTA	CCCAAGCTTCAGTCTCCCGA GTAGCTG
-802/+100nt	CTAGCTAGCAGGCACAAGAATGGCCTG	CCCAAGCTTTAAACTAACCT TACCTGT
-1199/-803nt	CTAGCTAGCTATTGTATTCAGTGGCTG	CCCAAGCTTCTCAGTCTCCC GAGTAGC
-1500/-1200nt	CTAGCTAGCTGTCTTAGAGGGTGCTTA	CCCAAGCTTAATATGGTAGA GGAAAGG
Sp-1 mutant upper part	CTAGCTAGCTATTGTATTCAGTGGCTG	CTCAAATGATCCACACGTCTAG GCCTCCCA
Sp-1 mutant lower part	TGGGAGGCCTAGACGTGTGG ATCATTTGAG	CCCAAGCTTCTCAGTCTCCC GAGTAGC
**Primers for CDS cloning**		
C4BPα	CCCAAGCTTATGCACCCCCCAAAAACTC	CCGCTCGAGTTATAGTT CTTTATCC
**Primers for RT-PCR and qRT-PCR**		
C4BPα	ACCTTGATCGCTGCTCTGTT	TGGGTGTCTGCATCGTTTGT
β-actin	CTTAGTTGCGTTACACCCTTTC	CACCTTCACCGTTCCAGTTT
HBx	ATGGCTGCTAGGGTGTGCT	TAAATCTCCTCCCCCAACTC
18SrRNA	CGGCTACCACATCCAAGGAA	GCTGGAATTACCGCGGCT
GAPDH	ATCACCATCTTCCAGGAGCGA	CCTTCTCCATGGTGGTGAAGAC
**Primers for ChIP**		
C4BP Chip	GAAAGGCACTTACAAGAA	ACCTCAAATGATCCACCC
**siRNA sequences**		
siHBx	AAGAGGACUCUUGGACUCUCA	
siControl	UUCUCCGAACGUGUCACGU	
siC4BPα1	CCUGCUGUUCUUGGCAAUU	
siC4BPα2	CCUGCCAAUCACUGUGUUU	
siSp1	GCCAAUAGCUACUCAACUA	
