# Supplementary material for: Hepatitis B virus X protein up-regulates C4b-binding protein α through activating transcription factor Sp1 in protection of hepatoma cells from complement attack
Source: Oncotarget. 2016 Mar 30;7(19):28013–26. doi: 10.18632/oncotarget.8472 (PMC5053706; doi:10.18632/oncotarget.8472)
Supplement: Supplementary file 2 [file oncotarget-07-28013-s002.doc]

**Supplementary Table 1**. Clinical characteristics of liver cancer from microarray samples.

|  | **No.** | | **Age** | | **Sex** | **Organ** | | **Pathology diagnosis** | **Grade** |  | | |  |
| --- | --- | --- | --- | --- | --- | --- | --- | --- | --- | --- | --- | --- | --- |
| 1 | | 55 | | F | | | Liver | hepatocellular carcinoma | Ⅱ | |  | | |
| 2 | | 32 | | F | | | Liver | hepatocellular carcinoma | Ⅰ | |  | | |
| 3 | | 38 | | M | | | Liver | hepatocellular carcinoma | Ⅱ | |  | | |
| 4 | | 63 | | F | | | Liver | hepatocellular carcinoma | Ⅰ | |  | | |
| 5 | | 35 | | M | | | Liver | hepatocellular carcinoma | Ⅰ | |  | | |
| 6 | | 42 | | F | | | Liver | hepatocellular carcinoma | Ⅰ | |  | | |
| 7 | | 46 | | M | | | Liver | hepatocellular carcinoma | Ⅰ | |  | | |
| 8 | | 40 | | M | | | Liver | hepatocellular carcinoma | Ⅰ | |  | | |
| 9 | | 48 | | F | | | Liver | hepatocellular carcinoma | Ⅰ | |  | | |
| 10 | | 40 | | M | | | Liver | hepatocellular carcinoma | Ⅰ | |  | | |
| 11 | | 41 | | F | | | Liver | hepatocellular carcinoma | Ⅰ | |  | | |
| 12 | | 49 | | M | | | Liver | hepatocellular carcinoma | Ⅰ | |  | | |
| 13 | | 42 | | M | | | Liver | hepatocellular carcinoma | Ⅰ | |  | | |
| 14 | | 18 | | M | | | Liver | hepatocellular carcinoma | Ⅰ | |  | | |
| 15 | | 55 | | M | | | Liver | hepatocellular carcinoma | Ⅰ | |  | | |
| 16 | | 71 | | M | | | Liver | hepatocellular carcinoma | Ⅱ | |  | | |
| 17 | | 43 | | F | | | Liver | hepatocellular carcinoma | Ⅱ | |  | | |
| 18 | | 50 | | M | | | Liver | hepatocellular carcinoma | Ⅰ | |  | | |
| 19 | | 98 | | F | | | Liver | hepatocellular carcinoma | Ⅱ | |  | | |
| 20 | | 48 | | F | | | Liver | hepatocellular carcinoma | Ⅲ | |  | | |
| 21 | | 58 | | M | | | Liver | hepatocellular carcinoma | Ⅱ | |  | | |
| 22 | | 70 | | M | | | Liver | hepatocellular carcinoma | Ⅱ | |  | | |
| 23 | | 49 | | M | | | Liver | hepatocellular carcinoma | Ⅲ | |  | | |
| 24 | | 52 | | M | | | Liver | hepatocellular carcinoma | Ⅱ | |  | | |
| 25 | | 56 | | M | | | Liver | hepatocellular carcinoma | Ⅱ | |  | | |
| 26 | | 58 | | M | | | Liver | hepatocellular carcinoma | Ⅱ | |  | | |
| 27 | | 61 | | M | | | Liver | hepatocellular carcinoma | Ⅱ | |  | | |
| 28 | | 50 | | M | | | Liver | hepatocellular carcinoma | Ⅱ | |  | | |
| 29 | | 39 | | M | | | Liver | hepatocellular carcinoma | Ⅱ | |  | | |
| 30 | | 37 | | F | | | Liver | hepatocellular carcinoma | Ⅱ | |  | | |
| 31 | | 45 | | M | | | Liver | hepatocellular carcinoma | Ⅱ | |  | | |
| 32 | | 56 | | M | | | Liver | hepatocellular carcinoma | Ⅱ | |  | | |
| 33 | | 64 | | M | | | Liver | hepatocellular carcinoma | Ⅱ | |  | | |
| 34 | | 69 | | F | | | Liver | hepatocellular carcinoma | Ⅱ | |  | | |
| 35 | | 58 | | M | | | Liver | hepatocellular carcinoma | Ⅱ | |  | | |
| 36 | | 47 | | M | | | Liver | hepatocellular carcinoma | Ⅱ | |  | | |
| 37 | | 49 | | F | | | Liver | hepatocellular carcinoma | Ⅱ | |  | | |
| 38 | | 48 | | M | | | Liver | hepatocellular carcinoma | Ⅱ | |  | | |
| 39 | | 49 | | M | | | Liver | hepatocellular carcinoma | Ⅱ | |  | | |
| 40 | | 43 | | M | | | Liver | hepatocellular carcinoma | Ⅱ | |  | | |
| 41 | | 42 | | M | | | Liver | hepatocellular carcinoma | Ⅱ | |  | | |
| 42 | | 35 | | M | | | Liver | hepatocellular carcinoma | Ⅱ | |  | | |
| 43 | | 50 | | M | | | Liver | hepatocellular carcinoma | Ⅱ | |  | | |
| 44 | | 40 | | M | | | Liver | hepatocellular carcinoma | Ⅱ | |  | | |
| 45 | | 33 | | M | | | Liver | hepatocellular carcinoma | Ⅱ | |  | | |
| 46 | | 57 | | M | | | Liver | hepatocellular carcinoma | Ⅲ | |  | | |
| 47 | | 55 | | M | | | Liver | hepatocellular carcinoma | Ⅱ | |  | | |
| 48 | | 36 | | F | | | Liver | hepatocellular carcinoma | Ⅱ | |  | | |
| 49 | | 63 | | M | | | Liver | hepatocellular carcinoma | Ⅱ | |  | | |
| 50 | | 19 | | M | | | Liver | hepatocellular carcinoma | Ⅱ | |  | | |
| 51 | | 68 | | M | | | Liver | hepatocellular carcinoma | Ⅱ | |  | | |
| 52 | | 40 | | M | | | Liver | hepatocellular carcinoma | Ⅱ | |  | | |
| 53 | | 52 | | F | | | Liver | hepatocellular carcinoma | Ⅱ | |  | | |
| 54 | | 38 | | M | | | Liver | hepatocellular carcinoma | Ⅱ | |  | | |
| 55 | | 48 | | M | | | Liver | hepatocellular carcinoma | Ⅱ | |  | | |
| 56 | | 53 | | M | | | Liver | hepatocellular carcinoma | Ⅱ | |  | | |
| 57 | | 41 | | M | | | Liver | hepatocellular carcinoma | Ⅱ | |  | | |
| 58 | | 35 | | F | | | Liver | hepatocellular carcinoma | Ⅱ | |  | | |
| 59 | | 27 | | M | | | Liver | hepatocellular carcinoma | Ⅲ | |  | | |
| 60 | | 65 | | M | | | Liver | hepatocellular carcinoma | Ⅱ | |  | | |
| 61 | | 39 | | F | | | Liver | hepatocellular carcinoma | Ⅱ | |  | | |
| 62 | | 41 | | M | | | Liver | hepatocellular carcinoma | Ⅱ | |  | | |
| 63 | | 46 | | M | | | Liver | hepatocellular carcinoma | Ⅱ | |  | | |
| 64 | | 60 | | M | | | Liver | hepatocellular carcinoma | Ⅱ | |  | | |
| 65 | | 41 | | M | | | Liver | hepatocellular carcinoma | Ⅱ | |  | | |
| 66 | | 45 | | M | | | Liver | hepatocellular carcinoma | Ⅱ | |  | | |
| 67 | | 48 | | F | | | Liver | hepatocellular carcinoma | Ⅱ | |  | | |
| 68 | | 47 | | M | | | Liver | hepatocellular carcinoma | Ⅱ | |  | | |
| 69 | | 47 | | M | | | Liver | hepatocellular carcinoma | Ⅱ | |  | | |
| 70 | | 75 | | M | | | Liver | hepatocellular carcinoma | Ⅱ | |  | | |
| 71 | | 25 | | M | | | Liver | hepatocellular carcinoma | Ⅱ | |  | | |
| 72 | | 51 | | M | | | Liver | hepatocellular carcinoma | Ⅲ | |  | | |
| 73 | | 55 | | M | | | Liver | hepatocellular carcinoma | Ⅱ | |  | | |
| 74 | | 65 | | M | | | Liver | hepatocellular carcinoma | Ⅱ | |  | | |
| 75 | | 45 | | M | | | Liver | hepatocellular carcinoma | Ⅱ | |  | | |
| 76 | | 46 | | M | | | Liver | hepatocellular carcinoma | Ⅱ | |  | | |
| 77 | | 46 | | M | | | Liver | hepatocellular carcinoma | Ⅱ | |  | | |
| 78 | | 43 | | M | | | Liver | hepatocellular carcinoma | Ⅱ | |  | | |
| 79 | | 62 | | F | | | Liver | hepatocellular carcinoma | Ⅲ | |  | | |
| 80 | | 35 | | M | | | Liver | hepatocellular carcinoma | Ⅱ | |  | | |
| 81 | | 47 | | F | | | Liver | hepatocellular carcinoma | Ⅱ | |  | | |
| 82 | | 47 | | M | | | Liver | hepatocellular carcinoma | Ⅱ | |  | | |
| 83 | | 67 | | M | | | Liver | hepatocellular carcinoma | Ⅱ | |  | | |
| 84 | | 63 | | M | | | Liver | hepatocellular carcinoma | Ⅱ | |  | | |
| 85 | | 45 | | M | | | Liver | hepatocellular carcinoma | Ⅱ | |  | | |
| 86 | | 52 | | F | | | Liver | hepatocellular carcinoma | Ⅱ | |  | | |
| 87 | | 48 | | F | | | Liver | hepatocellular carcinoma | Ⅱ | |  | | |
| 88 | | 37 | | M | | | Liver | hepatocellular carcinoma | Ⅲ | |  | | |
| 89 | | 69 | | F | | | Liver | hepatocellular carcinoma | Ⅰ | |  | | |
| 90 | | 60 | | M | | | Liver | hepatocellular carcinoma | Ⅱ | |  | | |
| 91 | | 62 | | F | | | Liver | hepatocellular carcinoma | Ⅱ | |  | | |
| 92 | | 70 | | M | | | Liver | hepatocellular carcinoma | Ⅱ | |  | | |
| 93 | | 40 | | M | | | Liver | hepatocellular carcinoma | Ⅱ | |  | | |
| 94 | | 45 | | M | | | Liver | hepatocellular carcinoma | Ⅱ | |  | | |
| 95 | | 40 | | M | | | Liver | hepatocellular carcinoma | Ⅱ | |  | | |
| 96 | | 74 | | M | | | Liver | hepatocellular carcinoma | Ⅱ | |  | | |
| 97 | | 45 | | M | | | Liver | hepatocellular carcinoma | Ⅱ | |  | | |
| 98 | | 48 | | F | | | Liver | hepatocellular carcinoma | Ⅱ | |  | | |
| 99 | | 40 | | M | | | Liver | hepatocellular carcinoma | Ⅱ | |  | | |
| 100 | | 47 | | M | | | Liver | hepatocellular carcinoma | Ⅲ | |  | | |
| 101 | | 32 | | M | | | Liver | hepatocellular carcinoma | Ⅱ | |  | | |
| 102 | | 58 | | M | | | Liver | hepatocellular carcinoma | Ⅱ | |  | | |
| 103 | | 47 | | M | | | Liver | hepatocellular carcinoma | Ⅱ | |  | | |
| 104 | | 26 | | M | | | Liver | hepatocellular carcinoma | Ⅱ | |  | | |
| 105 | | 65 | | F | | | Liver | hepatocellular carcinoma | Ⅱ | |  | | |
| 106 | | 52 | | M | | | Liver | hepatocellular carcinoma | Ⅲ | |  | | |
| 107 | | 54 | | M | | | Liver | hepatocellular carcinoma | Ⅱ | |  | | |
| 108 | | 48 | | M | | | Liver | hepatocellular carcinoma | Ⅱ | |  | | |
| 109 | | 63 | | M | | | Liver | hepatocellular carcinoma | Ⅱ | |  | | |
| 110 | | 63 | | M | | | Liver | hepatocellular carcinoma | Ⅲ | |  | | |
| 111 | | 67 | | M | | | Liver | hepatocellular carcinoma | Ⅲ | |  | | |
| 112 | | 43 | | M | | | Liver | hepatocellular carcinoma | Ⅲ | |  | | |
| 113 | | 46 | | M | | | Liver | hepatocellular carcinoma | Ⅲ | |  | | |
| 114 | | 35 | | M | | | Liver | hepatocellular carcinoma | Ⅲ | |  | | |
| 115 | | 38 | | M | | | Liver | hepatocellular carcinoma | Ⅲ | |  | | |
| 116 | | 58 | | M | | | Liver | hepatocellular carcinoma | Ⅲ | |  | | |
| 117 | | 41 | | M | | | Liver | hepatocellular carcinoma | Ⅲ | |  | | |
| 118 | | 56 | | M | | | Liver | hepatocellular carcinoma | Ⅲ | |  | | |
| 119 | | 72 | | M | | | Liver | hepatocellular carcinoma | Ⅲ | |  | | |
| 120 | | 65 | | M | | | Liver | hepatocellular carcinoma | Ⅲ | |  | | |
| 121 | | 56 | | M | | | Liver | hepatocellular carcinoma | Ⅲ | |  | | |
| 122 | | 38 | | M | | | Liver | hepatocellular carcinoma | Ⅲ | |  | | |
| 123 | | 43 | | F | | | Liver | hepatocellular carcinoma | Ⅲ | |  | | |
| 124 | | 51 | | M | | | Liver | hepatocellular carcinoma | Ⅰ | |  | | |
| 125 | | 51 | | M | | | Liver | hepatocellular carcinoma | Ⅲ | |  | | |
| 126 | | 52 | | M | | | Liver | hepatocellular carcinoma | Ⅲ | |  | | |
| 127 | | 68 | | M | | | Liver | hepatocellular carcinoma | Ⅲ | |  | | |
| 128 | | 52 | | M | | | Liver | hepatocellular carcinoma | Ⅲ | |  | | |
| 129 | | 50 | | M | | | Liver | hepatocellular carcinoma | Ⅲ | |  | | |
| 130 | | 49 | | M | | | Liver | hepatocellular carcinoma | Ⅲ | |  | | |
| 131 | | 56 | | F | | | Liver | hepatocellular carcinoma | Ⅲ | |  | | |
| 132 | | 68 | | M | | | Liver | hepatocellular carcinoma | Ⅲ | |  | | |
| 133 | | 55 | | M | | | Liver | hepatocellular carcinoma | Ⅲ | |  | | |
| 134 | | 54 | | M | | | Liver | hepatocellular carcinoma | Ⅲ | |  | | |
| 135 | | 62 | | M | | | Liver | hepatocellular carcinoma | Ⅲ | |  | | |
| 136 | | 53 | | M | | | Liver | hepatocellular carcinoma | Ⅲ | |  | | |
| 137 | | 66 | | M | | | Liver | hepatocellular carcinoma | Ⅲ | |  | | |
| 138 | | 56 | | M | | | Liver | hepatocellular carcinoma | Ⅲ | |  | | |
| 139 | | 32 | | F | | | Liver | hepatocellular carcinoma | Ⅲ | |  | | |
| 140 | | 47 | | M | | | Liver | hepatocellular carcinoma | Ⅲ | |  | | |
| 141 | | 39 | | M | | | Liver | hepatocellular carcinoma | Ⅲ | |  | | |
| 142 | | 56 | | F | | | Liver | hepatocellular carcinoma | Ⅲ | |  | | |
| 143 | | 68 | | M | | | Liver | hepatocellular carcinoma | Ⅲ | |  | | |
| 144 | | 57 | | F | | | Liver | hepatic tissue | ─ | |  | | |
| 145 | | 38 | | F | | | Liver | hepatic tissue | ─ | |  | | |
| 146 | | 60 | | M | | | Liver | hepatic tissue | ─ | |  | | |
| 147 | | 46 | | M | | | Liver | hepatic tissue | ─ | |  | | |
| 148 | | 31 | | M | | | Liver | hepatic tissue | ─ | |  | | |
| 149 | | 63 | | F | | | Liver | hepatic tissue | ─ | |  | | |
| 150 | | 63 | | M | | | Liver | hepatic tissue | ─ | |  | | |
| 151 | | 35 | | M | | | Liver | hepatic tissue | ─ | |  | | |
|  |  | |  | |  |  | |  |  |  | |  | |

Note: “-” No grading available.
